# A systematic review on the impact of diabetes mellitus on the ocular surface

**DOI:** 10.1038/nutd.2017.4

**Published:** 2017-03-20

**Authors:** K Co Shih, K S-L Lam, L Tong

**Affiliations:** 1 Department of Ophthalmology, Li Ka Shing Faculty of Medicine, The University of Hong Kong, Hong Kong SAR, China; 2 Research Center of Heart, Brain, Hormone and Healthy Aging, The University of Hong Kong, Hong Kong; 3 Department of Medicine, The University of Hong Kong, Hong Kong; 4 State Key Laboratory of Pharmaceutical Biotechnology, The University of Hong Kong, Hong Kong; 5 Singapore Eye Research Institute, National Eye Centre, Singapore; 6 Yong Loo Lin School of Medicine, National University of Singapore, Singapore; 7 Department of Ophthalmology, Singapore National Eye Center, Singapore; 8 Department of Ophthalmology, Duke-NUS Medical School, Singapore

## Abstract

Diabetes mellitus is associated with extensive morbidity and mortality in any human community. It is well understood that the burden of diabetes is attributed to chronic progressive damage in major end-organs, but it is underappreciated that the most superficial and transparent organ affected by diabetes is the cornea. Different corneal components (epithelium, nerves, immune cells and endothelium) underpin specific systemic complications of diabetes. Just as diabetic retinopathy is a marker of more generalized microvascular disease, corneal nerve changes can predict peripheral and autonomic neuropathy, providing a window of opportunity for early treatment. In addition, alterations of immune cells in corneas suggest an inflammatory component in diabetic complications. Furthermore, impaired corneal epithelial wound healing may also imply more widespread disease. The non-invasiveness and improvement in imaging technology facilitates the emergence of new screening tools. Systemic control of diabetes can improve ocular surface health, possibly aided by anti-inflammatory and vasoprotective agents.

## Introduction

**Figure 1:**
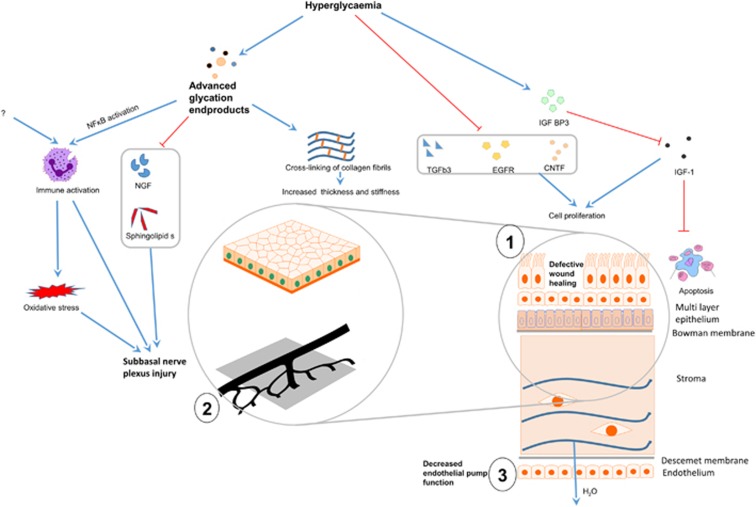
Schematic showing pathogenesis of corneal disease in diabetes mellitus. Hyperglycemia and formation of advanced glycation end products have distinct effects on different parts of the cornea, resulting in three principal types of tissue dysfunction with physiological effects that can be measured. (**1**) Defective wound healing in the corneal epithelium, (**2**) abnormalities of sub-basal nerves and (**3**) loss of corneal endothelial pump function. (**1**) Raised blood glucose promotes IGFBP3 release, which in turn competitively inhibits IGF-1, whereas TGFb3, EGFR, CNTF are suppressed in hyperglycemic states. The consequential reduction in epithelial cell proliferation and increased apoptosis impacts on epithelial wound healing. (**2**) Neuronal damage is a key defect in diabetes mellitus. Prolonged hyperglycemia results in the accumulation of advanced glycation end products which promotes inflammation and oxidative stress. NGF and sphingolipids are key to neuronal health and myelin formation, but their production are inhibited in hyperglycemic states. (**3**) Prolonged hyperglycemia also results in endothelial cell loss and impairment in pump function. Apart from these processes, the swelling of the corneal stroma (the main bulk of the cornea) may be due to loss of epithelial barrier, crosslinking of stromal collagen and matrix, and loss of the endothelial pump. CNTF, ciliary neurotrophic factor; EGFR, epithelial growth factor receptor; IGF-1, insulin-like growth factor 1; NF-kB, nuclear factor kappa-light-chain-enhancer of activated B cells transcription factor; NGF, nerve growth factor; TGFb3, transforming growth factor beta-3. Solid blue arrows—activation/promotion, red stop arrows—inhibition or negative regulation.

Diabetes mellitus (DM) is a significant public health problem. It is estimated that more than 342 million people worldwide will suffer from DM by 2030 and the total health burden incurred by DM will be driven by the severity of diabetic complications in different organs. The ocular surface, including the superficial and transparent cornea, is known to be involved in diabetes in various ways: this includes common diseases like dry eye and recurrent corneal erosions, previously reviewed elsewhere.^1^ However, new research beyond 2008 has not been systematically reviewed, even after the emergence of fairly recent review articles.^2, 3, 4, 5, 6, 7, 8, 9, 10, 11, 12, 13, 14, 15, 16, 17, 18, 19^ This is an important issue to address as new developments such as cellular, molecular biology and animal genetics have advanced considerably in the last few years. Here, we provide a systematic review of the recent literature (published 2009–2015), which enlightens on the role of the ocular surface and cornea in DM (Figure 1) and research on potential treatment strategies.

## Materials and methods

A literature search was conducted on the 5th of January 2016 in the NCBI Entrez Pubmed database and included search terms *diabetes* and *cornea*. Articles were limited to journal articles in which the keywords ‘cornea' or ‘conjunctiva' occur in conjunction with the keyword ‘diabetes' in the textword (tw) field of the search. We only examined journal articles published between 1st of January 2009 and 31st of December 2016. The 234 articles identified were then curated by two coauthors (KS and LT) for relevance, via abstract or the full text of the article, and this produced a list of 23 review articles, letters or commentaries (18 reviews) and 110 relevant original articles. For example, articles that involved only the posterior segment of the eye (retina, vitreous) or involving only ‘diabetes insipidus' and not ‘diabetes mellitus' would be considered irrelevant. A total of 106 were deemed irrelevant.

## Cornea epithelial disease and ocular surface abnormalities

It is known that diabetes is associated with impaired wound healing. This is evident in the corneal epithelium. Diabetic eyes are at increased risk of dry eye, superficial punctate keratitis, recurrent corneal erosion syndrome and persistent epithelial defects.^20, 21^ As the corneal epithelium is the first layer of the eye, it is constantly subjected to wear and tear and it needs to be constantly regenerated. Any process that affects wound healing or the speed of epithelial regeneration will have physiological impact and increases morbidity including ocular pain and redness.^22, 23, 24, 25^

Recently a human study conducted in a hospital showed for the first time that tear levels of type 1 and 2 diabetic individuals had significantly higher insulin-like growth factor binding protein (IGFBP3) compared with age-matched normal adults. IGFBP3 is a multifunctional protein that is known to play a negative regulatory role in IGF signaling by binding and sequestrating it, competing with its cellular receptor IGFR-1. In the wider context, IGFBP3 has been known to regulate insulin resistance, apoptosis as well as oxidative damage. The processes regulating the secretion of IGFBP3 from corneal epithelial tissue is not known, but in experiments with immortalized human corneal epithelial cells, it was found that high levels of glucose in the culture medium can induce the production of IGFBP3, suggesting that the hyperglycemia in patients may be the cause of the IGFBP3 upregulation. This paper did not examine other potential sources of IGFBP3 such as lacrimal glands and immune cells. In addition, the clinical examination results of the corneal epithelium of the participants such as presence of epitheliopathy were not reported.^26^

Two studies with C57 db/db mice^27, 28^ and four studies involving rats^24, 29, 30, 31^ showed that hyperglycemia induced detrimental effects on the cornea epithelium-basement membrane complex. In these studies, decreases in corneal epithelial function were documented by an increase in corneal thickness, and structural changes were examined by electron microscopy.^24, 29, 30^ Since the normal corneal epithelium plays an important barrier function in excluding water from entering the stroma, a reduction in the barrier function will manifest as edema and swelling of the normally relatively dehydrated stroma. The component of the epithelium forming the barrier is largely sub-served by tight junctional complexes between cornea epithelial cells, visualized as electron dense structures. Loss or disruption of these tight junction structures or loss of basal corneal epithelial cells on imaging would explain loss of epithelial function. The loss of epithelial function can affect vision because the onset of edema causes cornea opacification and will directly affect transmission of light through the cornea.^32^

Use of diabetic animals has both advantages and constraints. The obvious advantage is the ability to obtain ocular tissue, but the main advantage of using these models is the possibility of examining changes before and after induction of diabetes. Such changes are almost impossible to evaluate in human patients as they would not have come to the attention of health professionals prior to the development of diabetes. The major limitation of animal models is that the induction of DM occurs fairly rapidly using one intervention compared with the more chronic, multi-factorial DM in humans, and therefore the disturbance may not be translatable to human disease. Induction of diabetes may be via injection of streptozotocin in Sprague Dawley (SD) rats, or in some cases, rats may be naturally diabetic such as Otsuka Long-Evans Tokushima fatty (OLETF) rats. Streptozotocin destroys beta islet cells in the pancreas of the animals, with reduction of insulin secretion and consequent hyperglycemia. The hyperglycemia-related effects as well as the oxidative stress associated with ingestion of fat and ethanol in OLEFT rats then induce systemic organ damage.^22^

One of the molecular changes detected in animal eyes is the measurement of levels of advanced glycation end products (AGE).^30^ This is an important pathological outcome as it is considered to be the mediator for all chronic DM complications including macrovascular and microvascular complications such as diabetic retinopathy in the eye. The accumulation of AGE in the cornea epithelium-basement membrane shifts local cell signaling towards pro-apoptotic and antiproliferative pathways, as well as increases oxidative stress and inflammation. A typical way of measuring oxidative stress in the ocular surface tissue is the quantification of the level of the oxidized nucleotide 8-hydroxydeoxyguanosine.^30^ A separate study found the gamma-glutamyl transferase level in the tear to be reduced in 14 type 1 and 2 DM participants compared with 14 control participants. However, in cornea buttons of cadavers, the tissue levels of the enzyme were lower in type 1 DM than in type 2 DM and controls. While these are interesting results in view of the possible role of the transferase in oxidative stress, more studies are required to determine the mechanistic significance.^33^

As DM is a chronic disease, it is important to differentiate molecular changes that induce the disease as opposed to molecules that mediate secondary complications. Distinct ultrastructural tissue alterations may occur in pre-diabetic eyes as well as in established DM.^29^ Here *in vitro* studies^23, 28^ and animal models^30^ are useful to chart the temporal changes as the corneal levels of epithelial growth factor receptor, ciliary neurotrophic factor and nuclear factor kappa B may be determined at different stages of disease. TGFb3, epithelial growth factor receptor and ciliary neurotrophic factor have already been found to promote corneal epithelial wound healing in studies on wound healing and found to be reduced in corneas of diabetic animals. The nuclear factor kappa B on the other hand is an important transcription factor that affects inflammation and cell development found to be increased in corneas of diabetic animals.^34, 35, 36^

## Cornea neuropathy

The sensory innervation of the cornea is a major determinant of epithelial health and healing capacity.^37^ This may be mediated by secretion of substance P by the nerves and binding to neurokinin-1 receptor on the epithelial cells.^38^ Corneal nerves are branches of the ophthalmic nerve, which is a branch of the trigeminal cranial nerve. They perforate the corneal stroma at the medial and lateral positions and branch into neurites that eventually sprout nerve endings anteriorly into the corneal epithelium.^3^

The cornea is the most densely innervated structure in humans, with nerve fibers playing an important neurotrophic role in the development of a healthy corneal surface. Loss of neurotrophic function may result in a non-healing or persistent cornea epithelial defect or neurotrophic ulcer. This has associated cornea edema and disturbance of visual function and is an important cause of morbidity in cornea clinics.^39^

Unlike other areas of the body, corneal nerves can be easily visualized in the transparent anterior corneal stroma by modern imaging techniques in clinical scenarios without invasive biopsy procedures. Essentially the *in vivo* findings have been confirmed by cadaveric *ex vivo* studies.^40^

### Advances in confocal imaging techniques

The most important advance in the last few years is the use of modern scanning laser ophthalmoscopy. The most common form of this *in vivo* confocal microscopy is the Heidelberg Retinal Tomography (Heidelberg, Germany), which is performed in conjunction with a corneal modular lens.^10, 11, 14, 41^ Images acquired are processed by imaging software for indices of nerve fiber density, nerve fiber length, nerve branch density and nerve tortuosity^42^ in the sub-basal nerve plexus because changes in this layer are more relevant in DM than in intrastromal nerves. One research group used NeuronJ, a plug-in for the NIH freeware Image J^43^ whereas the other group used proprietary ACModule and CCModule software developed in the University of Manchester.

Specific nerve indices may have been found to be useful in a particular region of the cornea for some clinical scenarios. Analysis of the sub-basal nerve plexus^44, 45, 46, 47, 48, 49^ can be performed in two regions of the cornea: central cornea and the inferior whorl.^50^ For example, it has also been reported that the nerve fiber density at the inferior whorl region is more sensitive to early nerve fiber damage than the central corneal region, in DM patients before development of peripheral neuropathy.^51^

Scans can be evaluated manually (CCModule), in semiautomated fashion (NeuronJ) or in a fully automated (ACCModule) technique.^52, 53^ All three methods were reported to have high repeatability, which can be further improved with experience but not by increasing magnification.^54, 55, 56, 57, 58, 59, 60^ The speed of image analysis can be improved with use of automated quantification techniques as well as wide-field imaging.^13, 61, 62^ Calculations from full automation are well correlated to those by manual methods, and so may be useful in communities without a manual evaluator.^5^

### Clinical studies

It is well known for many years that corneal nerve density is reduced in type 1 DM.^3^ Recently both types of DM have been associated with reduction of corneal nerve density and other corneal nerve abnormalities (Table 1).^19, 45, 47, 63, 64, 65^

Reduction in corneal nerve fiber density is a characteristic manifestation of diabetic corneal neuropathy, with demonstrated progression over time, in a 4 year cohort study of DM participants from two countries (Australia and UK).^66^

An interesting question would be whether good glycemic control restores the corneal nerve innervation and dysfunction? Two clinical cohort studies have concluded that once DM (type 1 and type 2) was established, good glycemic control was able to improve but not completely reverse corneal neuropathy.^67, 68^

Corneal nerve parameters have been found to correlate to diabetic peripheral neuropathy and autonomic neuropathy (Table 2).^43, 46, 48, 49, 66, 69, 70, 71, 72, 73^ Diabetic peripheral neuropathy, a common complication in up to 54% of diabetic population, is a significant cause of morbidity and poor quality of life in diabetic patients. As such, early detection of high-risk patients can pre-empt the course of the disease with measures such as better foot-care to improve healthcare outcomes.

Conventional clinical diagnosis of diabetic peripheral neuropathy includes clinical assessment and nerve conduction studies. However, these tests detect large fiber deficits, rather than the small unmyelinated C and thinly myelinated Aδ-nerve fibers which are affected earlier in the course of the disease.^15, 74, 75, 76^ The higher density and the preponderance of small nerve fibers in the cornea may explain why corneal nerve fiber changes can be detected before awareness of diabetic peripheral neuropathy in the lower limbs.^6, 16, 17, 45, 77^ Decreases in nerve fiber length in sub-basal nerve plexus have also been found to be associated with subclinical diabetic autonomic neuropathy which may be life-threatening, including cardiovascular complications such as arrhythmias or sudden cardiac deaths.^46, 71, 72^ The vagal function is used as a measure of autonomic neurological function, assessed by the change of heart rate in response to breathing and posture.

### Mechanisms of corneal neuropathy

Broadly, peripheral neuropathies are considered microvascular DM complications as a result of nerve ischemia. The conventional view is that AGE initiates damage to the pericytes and endothelium of capillaries and reduces microvascular supply to Schwann cells or neurons. This consequently decreases neuronal function. If the status of corneal nerves reflects peripheral nerve status, understanding the mechanism of the corneal neuropathy is vital.^3^

Microvascular abnormalities occur in the retina as well as in the cornea. Reduction of corneal nerve fiber density or length have been shown to predict the development of diabetic retinopathy as well as sight-threatening retinopathy.^45, 47, 78^

The formation of AGE may not be the initial trigger for pericyte damage or neuronal loss. Inflammation may play a role as high concentrations of Langerhans cells and dendritic cells, the main antigen-presenting cells in the ocular surface, aggregate around corneal nerve fibers early in the disease process.^79, 80^ In addition, levels of neurotrophic factors may be reduced in DM, for example, reduction in serum nerve growth factor and lipids such as sphingolipids have been detected in diabetic eyes compared with controls.^81, 82^

The cause or extent of immune dysfunction, if any, in humans with type 2 DM is not known. It has been found that immune cell infiltration of animal corneas may precede the induced hyperglycemia (ARVO 2015 e-abstract 3076). In fact, in a longitudinal rat study, the pre-diabetic obese rats have already manifested a similar amount of corneal nerve abnormalities with the diabetic rats.^67^ This suggests that accumulation of AGE, which is dependent on hyperglycemia and not present in pre-diabetic animals, is unlikely to be the cause of immune dysfunction in these animals. More animal studies with a mechanistic approach would be necessary to determine the cause of immune dysfunction in DM.

Do changes in corneal nerve structure lead to functional alteration? To answer this question the newer studies in Tables 1 and 2 also included corneal esthesiometry. This is a method of evaluation of corneal sensitivity which is a functional outcome of nerve innervation. These studies, except one, employed Cochrat Bonnet esthesiometry, which may not be as sensitive as a gas or Belmonte esthesiometry. Nevertheless, the studies were able to find an association between corneal nerve parameters and corneal sensitivity.^6, 46, 49, 75, 76, 79^

It is also well known that apart from being neurotrophic, loss of corneal sensation also reduced lacrimal tear production since the corneal receptors are the afferent limb of the lacrimal reflex arc.^3^ The reduction of corneal innervation has been linked to abnormal tear function as well as more frequent and severe symptoms of dry eye in DM patients.^9, 21, 27, 43, 83, 84, 85, 86^ These studies included those with or without previous surgical procedures such as LASIK and cataract surgeries. In one study, abnormal corneal innervation manifested as tear film dysfunction and debilitating, chronic irritation of the eye in type 1 but not in type 2 DM.^43^

## Cornea stroma and biomechanics

The corneal structure underneath the epithelium and the Bowman's layer is called the stroma. The corneal stroma is important because it accounts for 90% of the thickness of the cornea and therefore its tensile strength and biomechanical properties in general.^87^ The thickness of the human cornea is the most frequently measured parameter in clinical biometry of the eye. The main novelty in the recent clinical papers on corneal biomechanics involved the assessment of corneal hysteresis and resistance force, due to the recent availability of the Ocular Response Analyser,^84, 88, 89^ which can quantify these two parameters. Hysteresis refers to the amount of force required to indent the cornea as well as the recovery from the indentation. Resistance force is the hysteresis normalized to the cornea shape. A higher hysteresis suggests a more rigid and less deformable cornea. Seven cross-sectional clinic-based studies in Western Europe,^87, 90, 91, 92^ United States,^93^ Brazil,^89^ Israel^88^ and Iran^94^ have found type 1 and type 2 DM participants to have higher hysteresis, whereas only one study (Turkish study)^95^ found them to have a lower hysteresis, compared with age-matched controls. In one study, it was found that the fasting blood glucose level was significantly but weakly correlated (*r*=0.28) to the corneal hysteresis.^91^

Two other studies published in this recent period on corneal morphology found DM to be associated with a greater corneal thickness,^96, 97, 98^ which was consistent with reports earlier than 2008. It is noteworthy that patients with proliferative, non-proliferative retinopathies and those with no diabetic retinopathies did not have significantly different corneal thicknesses.^90, 97, 98, 99, 100^ In addition, a study in 100 children aged 6–17 years with type 1 DM in Romania had an increase in corneal thickness compared with an equivalent number of children of the same age,^101^ and similar findings had been reported in Turkey.^96^

The reason why DM is associated with greater corneal hysteresis or thicknesses is not completely known, apart from the relationship between increased corneal thicknesses in cases of overt corneal epitheliopathy. However, it has been speculated that the accumulation of AGE in the cornea stroma of diabetics may occur together with non-enzymatic crosslinking between collagen molecules and proteoglycans. The crosslinking would theoretically explain stiffening and thickening of the cornea. One study compared eight monkeys with insulin-dependent diabetes (streptozotocin injection) to four controls. In the diabetic eyes crosslinking has manifested ultra-structurally as abnormal collagen fibril aggregates in the stromal matrix on transmission electron microscopy.^102^ This is consistent with published evidence demonstrating AGE-induced crosslinking of extracellular matrix in diabetics, resulting in increased arterial stiffness.^103^ The fact that corneal thicknesses are elevated in children with DM who did not have other DM complications suggest that the cornea may be affected by AGE earlier than other organs.^96, 101^ Nevertheless, it is premature at present to speculate if corneal pachymetry, commonly done in eye clinics, can be used to detect early DM changes.

Given that AGE-related crosslinking of corneal proteins can change the shape or morphology of the cornea in DM, is it possible that DM may influence specific eye disorders that manifests with corneal biomechanical changes? One such example is keratoconus, a degeneration of the cornea characterized by progressive ectasia or thinning of the cornea, typically presenting at teenage or early adult years.^104^ Unfortunately, the published cross-sectional studies did not demonstrate a consistent association between DM and such alteration of corneal shapes.^93, 94^

## Cornea endothelial disease

Apart from the epithelium, the innermost layer of the cornea, called the corneal endothelium, plays a vital role in keeping the stroma dehydrated. This is because of the active pumping action of fluid from the cornea to the anterior chamber by the corneal endothelial cells. Similar types of regulated fluid transport are extremely important in diabetes in other contexts, for example, in the kidney.

Seven papers related to the corneal endothelium in DM have been published in the review period and all were hospital-based studies, except for one study comparing endothelial counts between diabetic and non-diabetic cadaveric donors.^105, 106, 107, 108, 109, 110, 111, 112^ The biggest study of the 5, conducted in Vellor, India, involved 153 participants with DM and 163 age-matched controls, and was performed on patients before and after cataract surgery up to 3 months postoperatively.^112^ Preoperative examinations showed no statistically significant difference between the groups in any of the corneal endothelial parameters. Both DM and controls had decreases in endothelial counts and increase in morphological abnormalities (increase in cell sizes or polymegathism and increased variability of shape called pleomorphism) at 6 weeks and 3 months post-operation. The authors reported that in the control group the rate of loss of corneal endothelial cells between 6 weeks and 3 months was relatively milder compared with the DM group (*P*=0.023). However, the actual measurements were not significantly different at any time points, suggesting that none of the differences discovered were clinically relevant. It is worth noting that this Indian study evaluated only small incision manual cataract surgery but did not investigate phacoemulsification; the latter is the more common form of surgery in the developed world and potentially induces more corneal endothelial cell loss than manual surgery.^113^

The other five papers were cross-sectional studies conducted in Korea, Malaysia and Hungary, Poland and Denmark.^105, 106, 107, 108, 109^ These studies excluded participants with prior cataract surgery or history of ocular disease, and reported statistically significant association of type 2 DM with increased clinical features of corneal endothelial dysfunction (reduced endothelial count, and polymegathism and pleomorphism). Nevertheless, the magnitude of the reported differences between the DM and age-matched controls in these studies was very small. For example, in the Malaysian study, the mean corneal endothelial counts was 2541 cells per mm^2^ in DM compared with 2660 cells per mm^2^ in controls, with a difference of about 120 cells per mm^2^. By excluding participants with ophthalmic problems, these studies would have included only participants with shorter duration of DM. Had these studies recruited DM participants with longer durations, it may be possible to discover greater magnitudes of differences.

## Advances in treatment of diabetic ocular surface

Systemic treatment in DM is the cornerstone of treatment in any diabetic complication. Tight blood glucose control, preferably in collaboration with an endocrinologist, can prevent further progression of corneal epitheliopathy and neuropathy.^63, 114^ Insulin treatment in diabetic mice reduced the level of oxidative stress in the lacrimal gland, assessed by total tissue peroxidase and malonaldehyde levels.^83^ The newer therapeutics approaches proposed in recent years and their limitations are summarized in Box 1.

The aim of local treatment in diabetic keratopathy is to maintain a smooth and lubricated ocular surface with an intact epithelium and adequate blink response. This minimizes visual distortion and maximizes comfort. The exact treatment prescribed is dependent on the severity of the problem and the specific structures involved. Early or mild disease will present as dry eye or recurrent erosions, and more severe disease in the form of neurotrophic ulcers and secondary infections. A step-wise approach towards treatment, such as that mentioned in the Dry Eye Workshop (DEWS), is helpful, aiming to halt further damage, encourage re-epithelialization, prevent infection and maintain adequate lubrication of the ocular surface.^2^

A previous review has already described how therapies like lubricants, antibiotics, autologous serum and anti-inflammatory agents, as well as devices, such as bandage contact lenses in DM patients.^2^ The benefit of autologous serum is that it contains growth factors that may further enhance epithelial wound healing. An irregular ocular surface may benefit from a bandage contact lens to reduce further trauma. For more severe conditions including neurotrophic ulcers, surgical options to induce eyelid closure, including botulinum toxin injection and tarsorrhaphy, may be required.

Some of the newer treatment modalities directed to the ocular surface have been recently reviewed are summarized in Box 2. The challenge of maintaining therapeutic concentrations of any topical drugs on the ocular surface is the rapid dilution by resident tears and elimination through the nasolacrimal duct.

## Conclusion and future studies

The assessment of the diabetic ocular surface has implications beyond eye care. Challenges and future directions in this field are described in the Boxes 1 and 2. The corneal nerve parameters are all age related and, therefore, widespread use of these features for screening patients will only be useful if age-stratified normative values are available for the target population.^17^ Reading centers for corneal imaging will play a major role in such initiatives. Use of special imaging techniques such as 2-photon microscopy in genetically modified mice with visible corneal nerves will be immensely valuable to investigate changes in animals with diabetic neuropathy, especially in the cornea.^7^

Apart from more conventional approaches, newer therapeutic agents including targeted molecular therapy, gene and stem cell therapies are promising but have not yet been translated to routine care. The bulk of the published work in these areas concerns evaluation in animal models and not clinical trials.

The future of diabetes management is dependent on increased awareness of the importance of the ocular surface in diabetes. An improved understanding of the ocular surface among the general medical profession is essential for optimal management.

## Figures and Tables

**Table 1:** Studies comparing corneal nerve parameters in diabetic subtypes

*Source*	*Country*	*Groups*^a^	*Sample size*	*Method*	*Parameter (mean)^b^*	*Outcomes*^c^	*Associations*
Messmer *et al.* (2010)^45^	Germany	Type I and II DM vs controls	13/54/24	HRT II Image J Esthesiometer	NFD (no. mm^−2^) NFL (mm mm^−^^2^) NBD (no. mm^−^^2^) NT	DM1/DM2/C: 16.9/16.1/23.3 9.7/10.7/16.1 1.5/1.6/1.4	Increasing severity of nerve fiber parameters with higher stages of diabetic retinopathy, history of nephropathy, peripheral neuropathy, and decreased corneal sensation predictive of abnormal CCM parameters, first paper to demonstrate abnormal CCM parameters in patients with normal corneal and vibration sensation
Ischibashi *et al.* (2012)^63^	Japan	Type I DM vs controls	38/38	HRT III Image J	NFD (no. mm^−^^2^) NFL (mm mm^−^^2^) NT Beading (mm)	DM1/C: 25.32/36.62 9.80/13.65 3.13/1.74 22.38/30.44	HbA1c level and blood pressure were an independent negative predictors of NFL and NFD
Nitoda *et al.* (2012)^47^	Greece	Type II DM (noDR/NPDR/NPDR/PDR) vs controls	46/47/46/47	HRT II, MATLAB	NFD (no. mm^−^^2^) NBD (no. mm^−^^2^) NFL (mm mm^−^^2^) NT	DM2 noDR/NPDR/PDR/C: 27.4/23.7/18.8/31.3 39.9/30.6/25/45.1 14.8/12.3/10.4/16.6 1.8/1.9/1.9/1.7	Positive correlation between corneal neuropathy and peripheral neuropathy
Zhivov *et al.* (2013)^64^	Germany	DM vs controls	36/20	HRT II GIMP Non-invasive esthesiometer	NFD (mm mm^−^^2^) NFL (mm mm^−^^2^) NBD (no. mm^−^^2^)	DM/C: 0.006/0.020 6.22/19.99 25.3/141.9	No difference in CCM parameters between patients with or without diabetic retinopathy, corneal sensation was significantly lower in the diabetic group than in controls
Wang *et al.* (2014)^19^	China	Type II DM vs controls	45/50	—	NFL (mm mm^−^^2^) NBD (no. mm^−^^2^) NT	DM2/C: 11/13 47/62 3.2/2.8	Pain severity of diabetic peripheral neuropathy showed negative correlation with NFL and NBD, positive correlation with NT
Ziegler *et al.* (2014)^65^	Germany	Type II DM vs controls	86/48	HRT II — Esthesiometer	NFL (mm mm^−^^2^) NFD (no. mm^−^^2^) NBD (no. mm^−^^2^)	DM2/C: 19.7/24.9 299.2/397.3 165.2/226.7	

Abbreviations:

a DM, diabetes mellitus; DR, diabetic retinopathy; NPDF, non-proliferative diabetic retinopathy; PDR, proliferative diabetic retinopathy.

b NBD, nerve branch density; NFD, nerve fiber density; NFL, nerve fiber length; NT, nerve tortuosity.

c C, control; DM1, type 1 diabetes mellitus; DM2, type 2 diabetes melliltus.

**Table 2:** Studies comparing corneal nerve parameters in different stages of diabetic peripheral neuropathy

*Source*	*Country*	*Groups*^a^	*Sample size*	*Method*	*Parameter (mean)*^b^	*Outcomes^c^*	*Associations*
Edwards *et al.* (2012)^69^	UK and Australia	Type I DM (without PN/with DPN) vs controls	143/88/61	HRT III CCMetrics	NFL (mm mm^−^^2^) NBD (no. mm^−^^2^) NT	DM1/C: 18.3/16/20 69/58/80 0.22/0.23/0.21	Baseline findings of longitudinal study: NFL and NBD strongly correlated with nerve conduction study parameters, NFL inversely correlated with HbA1c and duration diabetes
Petropoulos *et al.* (2013)^48^	UK	DM (no PN/mild PN/mod PN/sev PN) vs controls	50/26/17/18/47	HRT III CCMetrics	NFD (no. mm^−^^2^) NBD (no. mm^−^^2^) NFL (mm mm^−^^2^) NT	noPN/modPD/sev PN/C: 26.9/23.25/18.9/13.1/36.95 55.5/48.25/32.4/19.6/96.55 20.05/17.6/14.7/9.75/27.25 18.2/21.2/18.45/16.45/16.4	Symmetrical reduction in CCM parameters for all groups except those with severe neuropathy
Pritchard *et al.* (2014)^49^	UK and Australia	Type I DM (without PN/with DPN) vs controls	166/76/154	HRT III CCMetrics Esthesiometry	NFL (mm mm^−^^2^) NBD (no. mm^−^^2^)	DM2/C: 19/13/23 60/40/80	Baseline of longitudinal study, reduction in corneal nerve fiber length already noted in DM patients without peripheral neuropathy, reduction in corneal sensitivity only in type I DM patients with peripheral neuropathy
Stem *et al.* (2014)^70^	USA	DM (no PN/mild PN/severe PN) vs controls	25/10/8/9	HRT II NeuronJ	NFL (mm mm^−^^2^)	noPN/midPN/severe PN/C: 15.1/18.5/12.5/20.7	
DeMill *et al.* (2015)^43^	USA	DM (no or mild PN/severe PN) vs controls	16/9/9	HRT II NeuronJ Esthesiometry	NFL (mm mm^−^^2^)	noPN/severe PN/C: 18/12/20.5	Tear osmolarity increases and NFL decreases with increasing severity of PN, DM patients had lower Schirmer's test values than controls, no differences in OSDI or VFQ-25 scores, TBUT and ocular surface staining between groups
Tavakoli *et al.* (2015)^71^	UK	DM (without AN/with AN) vs controls	15/19/18	Confoscan P4	NFD (no. mm^−^^2^) NBD (no. mm^−^^2^) NFL (mm mm^−^^2^)	noAN/AN/C: 35.70/48.26 21.24/30.09 7.08/9.74	CCM findings correlated significantly with autonomic symptoms (COMPASS and CASS)
Misra *et al.* (2015)^46^	New Zealand	Type I DM vs controls	53/40	HRT II analySIS 3.1 Esthesiometry	Sub-basal nerve density (mm mm^−^^2^)	DM1/C: 11/21.17	Negative correlation between corneal sensitivity and autonomic nerve function, 50% of patients with abnormal CCM findings had otherwise no evidence of peripheral or autonomic neuropathy
Maddaloni *et al.* (2015)^72^	Italy	Type I DM (without AN/with AN) vs controls	36/20	Confoscan 4, Image J	NFD (no. mm^−^^2^) NFL (mm mm^−^^2^) Beading (mm)	noAN/AN/C: 51.7/32.8/92 1.4/1.9/1.4 14.8/15.3/20.6	CCM findings lower in DM patients with autonomic neuropathy than those without
Dehghani *et al.* (2014)^66^	Australia	Type I DM (without PN/with PN) vs controls	147/60	HRT III, ACCMetrics	NFD (no .mm^−^^2^) NBD (no. mm^−^^2^) NFL (mm mm^−^^2^)	noPN/withPN/C: 18.3/16.3/22.3 24.2/23.7/35.1 16/15/18.1	Baseline (left). Prospective: significant annual reduction in nerve fiber density in PN group vs controls (−0.9 per mm^2^ per year vs −0.06 per mm^2^ per year) CCM findings correlated with peroneal nerve conduction velocity (*r*=0.38) and cold sensation threshold (*r*=0.40)
Chen *et al.* (2015)^73^	UK	Type I DM (without PN/with PN) vs controls	63/26	HRT III CCMetrics ACMetrics	NFD (no. mm^−^^2^) NBD (no. mm^−^^2^) NFL (mm mm^−^^2^) NFD (no. mm^−^^2^) NBD (no. mm^−^^2^) NFL (mm mm^−^^2^)	noPN/withPN/C: 28.3/16.9/36.8 56.1/48.2/56.1 20.2/14.8/26.7 22.6/13.5/31.3 26.2/15.4/44.6 13.4/8.8/17.7	Comparable diagnostic efficacy between confocal microscopy measurements and intraepidermal nerve fiber density (via skin biopsy, gold standard)

Abbreviations:

a AN, diabetic autonomic neuropathy; DM, diabetes mellitus; PN, diabetic peripheral neuropathy.

b NBD, nerve branch density; NFD, nerve fiber density; NFL, nerve fiber length; NT, nerve tortuosity.

c C, control; DM1, type 1 diabetes mellitus; DM2, type 2 diabetes melliltus.

## New systemic therapies

- *Resolvin-D1*, an anti-inflammatory eicosanoid, reduced corneal and peripheral nerve degeneration in diabetic rats when administered as an oral supplement together with menhaden (fish) oil.^115^ The effect was independent of blood glucose levels.
- *Beta carotene*, an antioxidant, was shown to ameliorate diabetes-related ultrastructural changes to the cornea in a rat model.^116^ The beneficial effects of beta carotene was associated with a reduction in average blood glucose in treated groups.
- *Ilepatril,* a vasopeptidase inhibitor and new hypertension drug, can degrade vaso- and neuro-active petides as well as angiotension converting enzyme (ACE). In rats with streptozotocin-induced diabetes, oral administration of ilepatril protects against degeneration of the corneal nerves.^117^
- *Enalapril*, an ACE inhibitor, in combination with alpha lipoic acid (antioxidant) and menhaden oil was shown to reverse diabetic corneal and peripheral neuropathy in streptozotocin-induced diabetic rats when administrated per-orally.^118^
- *KIOM-79* (a mixture of 80% ethanol extracts of parched Puerariae radix, gingered Magnoliae cortex, Glycyrrhizae radix and Euphorbiae radix) can be used as an oral therapeutic agent by reducing AGE in tissues like the cornea. In a rat model of DM, it also reduced the downstream oxidative damage, nuclear factor kappa-B activation and Bax overexpression in the cornea.^119^
- *Experimental stem cell therapy*, in the form of human hematopoietic stem cells transplanted into the peritoneum of rats, reduced apoptosis in the corneal and conjunctival epithelium.^120^ However, the corneal nerves were not examined in those experiments. It is important to note that such xenograft approaches also have to deal with the expected immune response against transplanted cells.

## New topical therapies in diabetic ocular surface

- *Carnosine*, an antioxidant, may be used to counter the effects of AGE in the ocular surface.^115^ Topical administration of the substance has been shown to be effective in maintaining thiol levels in the cornea of rats with induced diabetes. This study unfortunately did not included assessment of the tear function and health of the ocular surface by imaging.^121^
- *Sericin* and *aloe vera* are topical protective agents and promote ocular surface wound healing.^122, 123^
- *Naltrexone*, a long-acting opioid antagonist, administrated topically or orally has been shown to accelerate corneal wound healing and restitute corneal sensitivity. It has also been shown to be safe and effective in more than one type of animal model.^12, 85, 86, 124, 125^
- Topical application of growth factors in diabetic animal models, in particular *insulin* and *nerve growth factor* (NGF), have had promising results.^81, 124^ Insulin is a much cheaper drug to produce than NGF, and therefore more likely to widely adopted, but it does not protect against the loss of corneal sensitivity. Moreover NGF can be easily degraded by alteration of temperature, pH and presence of tear proteases.^126^
- *Insulin-like growth factor 1* (IGF-1) promotes cell proliferation and when administrated topically to type 2 diabetic mice. Improved corneal sub-basal nerve density compared with controls.^127^
- Targeting microRNA *miR-146Aa* may be used to treat delays in wound healing in diabetic corneas, but this had been reported in organ cultures only.^128^
- *Experimental gene therapies* may be a viable form of local treatment for cornea disease in diabetics. Hepatic growth factor (HGF)-driven epithelial migration and wound closure is dependent on the function of the receptor tyrosine kinase c-Met. In DM animal models, the HGF levels were increased in the cornea epithelium, but the c-Met levels have decreased. Since inadequate c-Met levels may impair wound healing, adenoviral vector mediated overexpression of c-Met was attempted to correct the molecular anomaly. In organ-cultured human corneas from diabetic patients, the rate of epithelial migration was restored to the levels in the non-diabetic corneas. Though promising, such therapies have numerous hurdles to overcome, including safety concerns and issues related to the lack of specificity in delivery of expression vectors.^129, 130, 131^
